# Fatigue and Cognitive Fatigability in Mild Traumatic Brain Injury are Correlated with Altered Neural Activity during Vigilance Test Performance

**DOI:** 10.3389/fneur.2017.00496

**Published:** 2017-09-21

**Authors:** Marika C. Möller, Love Engström Nordin, Aniko Bartfai, Per Julin, Tie-Qiang Li

**Affiliations:** ^1^University Department of Rehabilitation Medicine, Danderyd Hospital, Stockholm, Sweden; ^2^Division of Rehabilitation Medicine, Department of Clinical Sciences, Karolinska Institutet, Stockholm, Sweden; ^3^Centre for Clinical Research, Sörmland, Uppsala University, Uppsala, Sweden; ^4^Department of Diagnostic Medical Physics, Karolinska University Hospital Huddinge, Stockholm, Sweden; ^5^Division of Clinical Geriatrics, Department of Neurobiology, Care Sciences and Society, Karolinska Institutet, Stockholm, Sweden; ^6^Division of Medical Imaging and Technology, Department of Clinical Science, Intervention and Technology, Karolinska Institutet, Stockholm, Sweden

**Keywords:** functional magnetic resonance imaging, mild traumatic brain injury, fatigue, pseudo-continuous arterial spin labeling, cerebral blood flow

## Abstract

**Introduction:**

Fatigue is the most frequently reported persistent symptom following a mild traumatic brain injury (mTBI), but the explanations for the persisting fatigue symptoms in mTBI remain controversial. In this study, we investigated the change of cerebral blood flow during the performance of a psychomotor vigilance task (PVT) by using pseudo-continuous arterial spin labeling (PCASL) MRI technique to better understand the relationship between fatigability and brain activity in mTBI.

**Material and methods:**

Ten patients (mean age: 37.5 ± 11.2 years) with persistent complaints of fatigue after mTBI and 10 healthy controls (mean age 36.9 ± 11.0 years) were studied. Both groups completed a 20-min long PVT inside a clinical MRI scanner during simultaneous measurements of reaction time and regional cerebral blood flow (rCBF) with PCASL technique. Cognitive fatigability and neural activity during PVT were analyzed by dividing the performance and rCBF data into quintiles in addition to the assessment of self-rated fatigue before and after the PVT.

**Results:**

The patients showed significant fatigability during the PVT while the controls had a stable performance. The variability in performance was also significantly higher among the patients, indicating monitoring difficulty. A three-way ANOVA, modeling of the rCBF data demonstrated that there was a significant interaction effect between the subject group and performance time during PVT in a mainly frontal/thalamic network, indicating that the pattern of rCBF change for the mTBI patients differed significantly from that of healthy controls. In the mTBI patients, fatigability at the end of the PVT was related to increased rCBF in the right middle frontal gyrus, while self-rated fatigue was related to increased rCBF in left medial frontal and anterior cingulate gyri and decreases of rCBF in a frontal/thalamic network during this period.

**Discussion:**

This study demonstrates that PCASL is a useful technique to investigate neural correlates of fatigability and fatigue in mTBI patients. Patients suffering from fatigue after mTBI used different brain networks compared to healthy controls during a vigilance task and in mTBI, there was a distinction between rCBF changes related to fatigability vs. perceived fatigue. Whether networks for fatigability and self-rated fatigue are different, needs to be investigated in future studies.

## Introduction

Fatigue is a frequently reported symptom after mild traumatic brain injury (mTBI) (1–3) and a major reason why patients fail to return to work (4). The subjective experience of fatigue may be concomitant with physiological fatigue or with deteriorating performance, but may also be a sole complaint (5, 6). Research on the relationship between underlying neural correlates to fatigue in mTBI, and possible performance decrements is complicated by the fact that fatigue is still not a well-defined concept. It is multidimensional in its nature, involving both physiological and psychological components (7–9) and, therefore, a single explanatory mechanism is unlikely (3, 10).

Kluger and coworkers (11) suggested distinguishing the self-rated fatigue measures from objective measures of fatigue by labeling the later as fatigability. Such distinction might encourage among others more focused correlational studies; such as fatigue in relation to the neural activity. Measuring performance during sustained cognitive process provides a method to evaluate fatigue/fatigability objectively (12–14). For example, sustained attention during vigilance performance is a demanding cognitive task and performance induced fatigability has been demonstrated as increased error rate and reaction time (15). Our group has also found fatigability in mTBI on a higher order attention demanding task (16).

More recently, we studied the behavioral correlates of changes in resting-state functional connectivity before and after performing a 20-min psychomotor vigilance task (PVT) for mTBI patients with persistent post-concussion fatigue (17). Taking advantage of a quantitative data-driven analysis approach developed by us, we were able to demonstrate that there was a significant linear correlation between the self-rated fatigue and functional connectivity in the thalamus and middle frontal cortex. Furthermore, we found that the 20 min PVT was sufficiently sensitive to invoke significant mental fatigue and specific functional connectivity changes in mTBI patients. These findings indicate that resting-state functional MRI (fMRI) measurements before and after a 20 min PVT may serve as a useful method for objective assessment of fatigue level in the neural attention system. However, these measurements neither provide any information about the dynamic change of the neural activities in the involved functional networks during the performance of PVT nor can they answer whether other neural systems mediate the observed functional connectivity change in the attention network.

Arterial spin labeling (ASL) MRI technique has recently been used to examine the cerebral blood flow (CBF) in patients with amnestic mild cognitive impairment and cognitively normal healthy controls both at rest and during the active performance of a memory task (18). As compared to rest, CBF measurement during the task performance showed increased group difference between patients and healthy controls indicating that CBF measures during a cognitive task may increase the discriminatory ability and the sensitivity to detect subtle functional changes in neurological diseases. In another ASL MRI study, Lim et al. (19) investigated the neural correlates of cognitive fatigue effects in a group of healthy volunteers during a 20-min PVT (19). They observed progressively slower reaction times and significantly increased mental fatigue ratings after the task and reported that such persistent cognitive fatigue effect was significantly correlated with regional cerebral blood flow (rCBF) decline in the right fronto-parietal attention network in addition to the basal ganglia and sensorimotor cortices. They also found that the rCBF at rest in the thalamus and right middle frontal gyrus before the PVT task was predictive of subjects’ subsequent performance decline. Based on these findings, they claimed that the rCBF at rest in the attention network might be a useful indicator of performance potential and a marker of the level of fatigue in neural attention system. However, it remains to be clarified how the relationship between the neural activity in mTBI patients and their fatigability is dynamically influenced by the performance of a difficult cognitive task.

Pseudo-Continuous Arterial Spin Labeling (PCASL) can provide quantitative rCBF measurements with whole-brain coverage and high signal-to-noise ratio. Furthermore, it is non-invasive and repetitive experiments can be carried out. It has been shown that fMRI experiments based on PCASL perfusion measurements may have higher sensitivity than experimental designs based on blood oxygenation level-dependent (BOLD) fMRI, particularly when studying slow neural activity changes within a subject (20–22) and useful as a biomarker of brain function (18). To shed light on the questions discussed above, in this study we used PCASL MRI technique to measure the rCBF changes during a 20 min PVT in a group of mTBI patients with chronic fatigue and matched healthy control subjects. The aims of the present study are the following: (1) evaluate the PVT induced fatigability over time by dividing the performance data (error rate and reaction time) into quintiles to verify if the change of fatigability for mTBI patients follows the same pattern as that for healthy controls; (2) estimate the dynamic change of neural activity during PVT in terms of rCBF measurements in each quintile to reveal brain activities significantly associated with the change of fatigability. (3) Voxel-wise assessment of the rCBF values pre- and post-PVT to detect brain activity associated with changes in self-rated fatigue level.

## Materials and Methods

### Participants

Ten patients (m/f 5/5) (age 18–55 years) with mild but persistent cognitive impairments after mTBI and with residual symptoms of fatigue [Fatigue Severity Scale (FSS) ≥4] were recruited to this study. All patients were in a chronic phase (more than 3 months after the injury), with a median of 5 years (range 0.5–9 years). They were referred for a neuropsychological evaluation due to persistent cognitive complaints. The mTBI was defined according to the criteria specified in Ref. (23). Exclusion criteria were: uncertain loss of consciousness (e.g., due to alcohol intoxication at the accident), signs of dementia indicated by CT-scan, subdural hematoma, hydrocephalus, or severe psychiatric or seizure disorder. Mean age was 37.5 years (SD 11.2), and mean length of education was 13.1 years (SD 1.6). Five patients suffered from fall accidents, three from traffic accidents, one patient had a horse riding accident, and one patient had a bicycle accident. A healthy control group (*n* = 10, m/f 5/5), matched for age, gender, and years of education on a group level, was also recruited to the study. The control group had a mean age of 36.9 years (SD 11.0), and the mean length of education was 13.4 years (SD 2.0). An experienced neuroradiologist evaluated the clinical MRI scans for all participants.

### The PVT

The reaction time paradigm (19) was created in E-Prime software (Psychology Software Tools Inc., Pittsburgh, PA, USA) as a sustained attention task. The participants were instructed to push a button as quickly as they could, with their dominant hand, when a set of four 0s appeared in a red rectangle, and do nothing if other numbers appeared. After each response, a visual feedback of the reaction time (RT) was displayed during 1 s. If the participant pushed the button at a false stimulus, or if the response time was more than 1 s, the feedback was “false answer” or “no answer,” respectively. The intervals between the stimuli varied 2–10 s in a pseudo-random fashion. The number of true positive responses varied somewhat (191–237 responses) between the individuals but the entire task always lasted 20 min. The number of RT responses from the PVT was divided into quintiles. For each individual, all quintiles included the same amount of responses but between the different individuals, the number of responses within each quintile could vary somewhat. Due to the random distribution of the stimuli the PVT quintile division was not as precise in time as the quintile division for the rCBF data. The mean reaction time (RT_mean_), SD (RT_Std_), and Relative SD (RT_RSD_) were calculated. RT_RSD_ was evaluated as the ratio between RT_Std_ and RT_mean_. The RT_RSD_ is also known as the coefficient of variance.

### Self-Rating Measures

The FSS was used to measure self-rated general fatigue. A mean score over 4 indicates fatigue (24, 25).

A Visual Analog Scale of Fatigue (VAS-f) was used to measure current self-rated fatigue level (“How fatigued do you feel right now”). The VAS-f ranged from 0 (corresponding to no fatigue) to 10 (corresponding to the worst possible fatigue).

A Swedish version of the Pittsburgh Sleep Quality Index (PSQI) was used to measure self-rated sleep quality (26).

Medications, tobacco, coffee and tea consumption, food and fluid intake, and the number of hours of sleep were noted. The participants were also asked if they had experienced anything that could have altered their responsiveness in proximity to the investigation.

### MRI Procedure

The participants completed the self-reported fatigue and sleep quality assessments before the MRI scanning. A 2-min long training session of the PVT preceded the MRI scans. All MRI data acquisition was conducted on a 3T Magnetom Trio MRI system (Siemens Medical Solutions, Erlangen, Germany) equipped with a 32-channel receive-only head coil. All data was acquired at Karolinska University Hospital Huddinge, Stockholm, between 12:00 noon and 5:00 p.m. to minimize circadian effects. The MRI acquisition protocol included a T1W MPRAGE, SWI, T2W GRE, T2W FLAIR, DTI, and three sessions of PCASL measurements before, during, and after the PVT. BOLD fMRI measurements were also conducted before and after the PVT, and the BOLD fMRI results have been published previously (17). In this report, we focus on the results from the PVT performance and the rCBF changes calculated from the PCASL measurements. The PCASL data was acquired using the following acquisition parameters; labeling duration = 1,600 ms, post-labeling delay = 1,200 ms, TE/TR = 18/3,330 ms, an FOV = 230 × 230, matrix size = 64 × 64, 18 slices of 6 mm thickness, interslice gap of 0.6 mm, a sampling bandwidth of 2,790 Hz/pixel, and an RF excitation flip angle of 90°. The PCASL data acquired at rest (before and after the PVT) lasted for 8:30 min:s (150 measurements), while the acquisition during the PVT lasted for 22:22 min:s (400 measurements).

The participants were carefully fixated in the head coil with paddings to reduce involuntary head motions. Before data acquisition, during resting-state conditions the participants were instructed to focus their sight on a white cross projected on a black screen and not to think about anything special. These instructions intended to keep the participants awake and minimize visual stimuli. The participants were in the MRI scanner for approximately 66 min. Following the MRI scanning, the participants once again rated their level of current fatigue on the VAS-f.

### Image Post-Processing

The post-processing of the PCASL data was performed off-line using shell scripts calling C-programs from the AFNI (Analysis of Functional NeuroImages, http://afni.nimh.nih.gov/afni/). The main steps included: (1) motion correction by 3D rigid-body image registration; (2) creation of brain mask; (3) voxel-wise rCBF computation according to previously established equation (27); and (4) brain normalization to align individual’s CBF image data to the Montreal Neurological Institute (MNI) brain template by using 12-parameter affine transformation and mutual information as the cost function.

### Statistics

Parametric methods were used when the data were on interval level and normally distributed. Independent Student’s *t*-test was used for comparison between patients and controls and paired Student’s *t*-test for comparison within subjects. Non-parametric methods were used for non-normally distributed variables and the self-rating measures. Mann–Whitney *U* test was then used for comparison between patients and controls, and Wilcoxon signed rank test for comparison within subjects. Depending on the type of data either Pearson correlation or Spearman’s rank was used for correlation analyses. Two-tailed *p*-values were used with a critical significance level of *p* < 0.05.

We used IBM SPSS Statistics (IBM Corp. Released 2013. IBM SPSS Statistics for Windows, Version 22.0. Armonk, NY, USA) for the statistical analysis of the PVT performance, demographic, and self-rating questionnaires. To identify the possible correlation between self-rated fatigue and fatigability measures (RT_mean_, RT_Std_, RT_RSD_), we also performed systematic regression analysis for the fatigue and fatigability data. Besides the self-rated VAS-f before and after PVT performance, the percentage change of VAS-f was also derived and used for the regression analysis.

The rCBF data calculated from the PCASL measurements were analyzed using a three-way ANOVA (AFNI, 3dANOVA3, model type = 5), regression analysis (AFNI, 3dRegAna) and additional *t*-tests (AFNI, 3dttest). The rCBF data during the PVT were normalized voxel-wise to each’s mean rCBF at rest before the onset of the task. The fixed factors of the three-way ANOVA are the time of PVT performance (df = 4, five quantiles of the PVT paradigm) and subject group (df = 1, mTBI patients vs. healthy controls). The individual subject (df = 9) was considered as a random factor. With three-way ANOVA, we can also assess the interaction between the two fixed factors and interrogate how the continuous PVT performance affected the local neural activity differently in mTBI patients and healthy volunteers.

Voxel-wise linear regression analysis was performed with rCBF image data using the AFNI program, 3dRegAna to study the possible correlation between fatigue and brain functional activity derived from rCBF measurements with PCASL. The data for all participants acquired during each quintile were first pooled together for the regression analysis to gain sufficient statistical power. Further regression analyses were also performed for each subject group separately. It should be noted that age and gender were included as cofactors to exclude possible confounding. The voxel-wise rCBF values from all participants were modeled as a linear function of their corresponding self-reported fatigue measures (VAS-f) post-MRI or cognitive fatigability regarding the mean (RT_mean_), SD (RT_std_) and relative SD (RT_RSD_) of RT measured during each quintile of the PVT performance. The regression analyses were performed by including either all participants or patients/controls separately.

The statistical significance at family-wise error rate (FWER), *p* < 0.05 for the three-way ANOVA and regression results were assessed first by setting a voxel-wise threshold at *p* < 0.01 and then by imposing a minimum voxel cluster size of at least 20 contiguous voxels. The minimum cluster size of 20 voxels was determined from the Monte-Carlo simulation results obtained by using the AFNI program, *AlphaSim*+, which was used to estimate the probability of the random field noise producing clusters of different sizes. For the simulations we used the following input parameters: matrix size = 64 × 64 × 18, a gray matter mask based on MNI template, voxel-wise threshold value *p* < 0.01, 10^6^ iterations, and Full-Width Half-Maximum (FWHM) = 6.8 mm, which was the estimated average by applying the AFNI program, 3dFWHMx, to the pooled rCBF image data, which was quite close to the FWHM value (6 mm) used in the final imaging smoothing procedure described above. The simulations with *AlphaSim* + produced a list of FWER *p*-values corresponding to the different cluster sizes, and a larger cluster size usually corresponds to a lower FWER *p*-value. For example, the corresponding FWER was *p* < 0.003, if imposing a cluster size of at least 30 contiguous voxels.

When multiple hypotheses are tested in statistical hypothesis testing as for our imaging data with multiple voxels in the brain, the likelihood of a rare event increases, which needs to be corrected. The most rigorous approach controlling for such false positives is to use the Bonferroni correction. The Bonferroni correction compensates for the increased likelihood in multiple comparisons by testing each hypothesis at a significance level of α/*N*, where α is the desired overall α level, and *N* is the number of hypotheses (the number of voxels in the brain in our case). The limitation of Bonferroni correction is that it assumes each voxel is independent. However, imaging data are not independent and furthermore are spatially smoothed. Therefore, Bonferroni correction was not carried out, as it tends to be overly conservative for controlling false positive findings and set the threshold too high to detect many true positives.

## Results

### Characterization of the Participants

There were no significant group differences regarding age and length of education, nor body mass index or caffeine consumption on the day of the MRI investigation. Self-rated sleep quality (PSQI) was approximately the same for the two groups (median_pat_ 9.5, range 2.0–14.0 vs. median_con_ 5.0 range 3.0–10.0, *p* = 0.063) but the patients slept longer compared to the controls the night before the MRI scanning (median_pat_ 8.0 h, range 6.0–10.0 h vs. median_con_ 6.0 h range 4.0–8.0 h, *p* = 0.013). No patient had structural pathological findings from the clinical evaluation of the MRI scans major enough to exclude them from the study, i.e., signs of contusion bleedings or subdural hematomas. One patient showed findings consistent with a diffuse axonal injury in the left hemisphere in the parasagittal frontal lobe and splenium as well as a suspected lesion in the pons on the right side. However, this patient did meet all inclusion criteria for mTBI and did not deviate on the PVT and was, therefore, included in the analyses. All patients were right-handed. Two controls were left-handed and six right-handed (two missing data). Every participant was instructed to use their dominant hand. One of the left-handed controls, preferred, despite left-hand dominance for writing, to push the button with the non-dominant hand. For those missing information, it was highly likely that they were right-handed because notifications were made if the participant departed from the standard procedure that the pressure device was held in the right hand.

### Fatigue Measures

**Figure 1:**
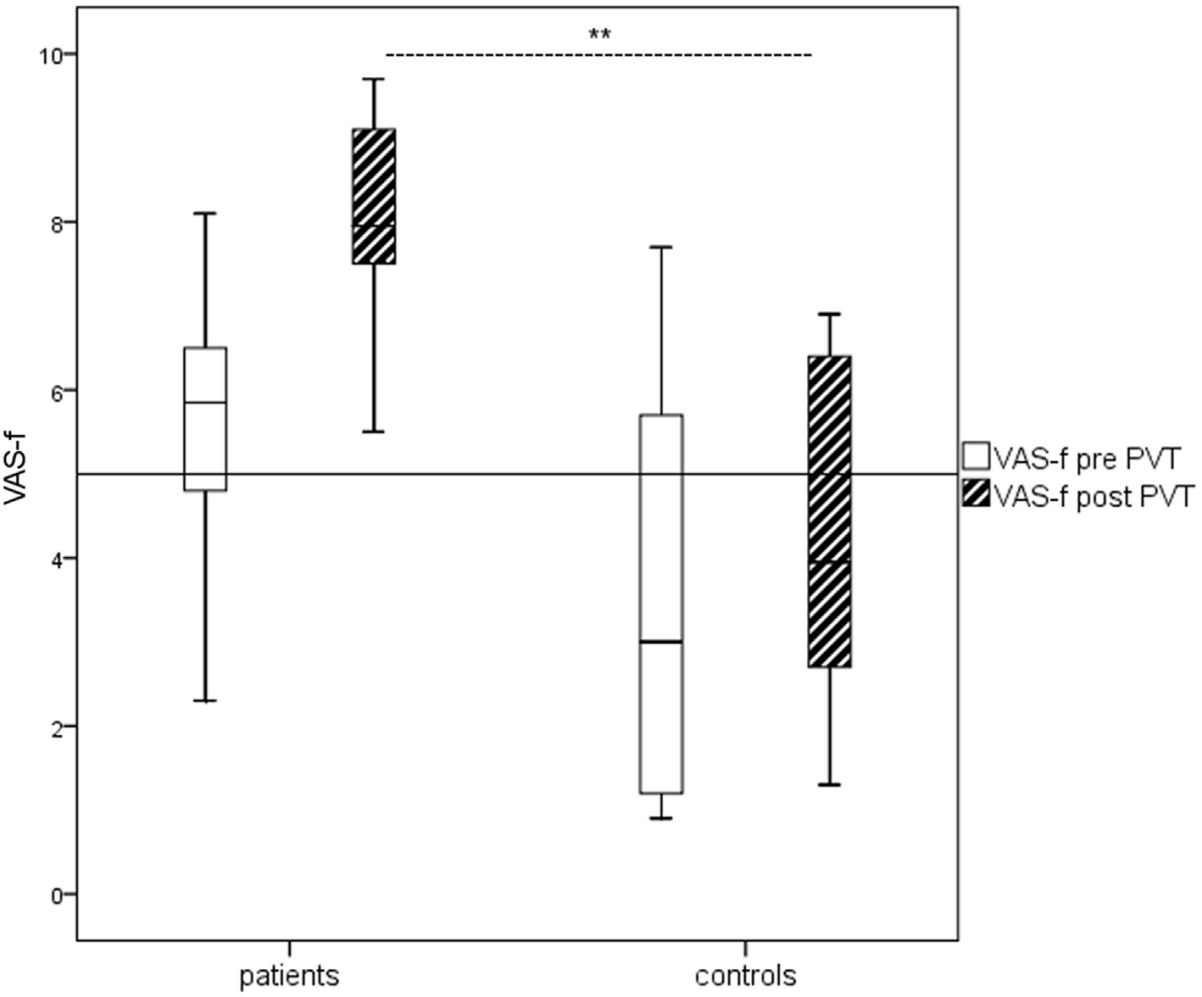
Boxplots of Visual Analog Scale of Fatigue (VAS-f) ratings before and after psychomotor vigilance task (PVT) for patients and controls, respectively. The mild traumatic brain injury patients reported significantly more self-rated current fatigue (VAS-f) than the healthy controls after the PVT (*p* < 0.01) according to Mann–Whitney *U* test.

The mTBI patients reported significantly higher general fatigue according to the FSS compared to the controls (median_pat_ 5.8, range 4.8–6.7 vs. median_con_ 2.6, range 1.8–3.0, *p* ≤ 0.001). Self-rated current fatigue (VAS-f) did not differ between the two groups before the PVT task but did so after the task (Figure 1). The number of hours of sleep the night before the investigation was not associated with the results on the FSS and VAS-f before the PVT according to Spearman rho.

### Performance on the PVT

The mean reaction time (RT_mean_) was significantly longer for the mTBI group (577 ± 162 vs. 414 ± 29 ms, *p* = 0.006). At the start of the PVT, only two patients performed within one SD of the mean for the controls. When RT was compared between the quintiles, the RT_mean_ per quintile increased for the patients but remained stable for the controls, and no overlap was observed between the groups from the second quintile onward. The mTBI patients also displayed significantly more scatter in the data, RT_Std_ (110 ± 47 vs. 61 ± 14 ms, *p* = 0.006) compared to the healthy controls. This can also be observed from the RT_RSD_ data (0.192 ± 0.057 vs. 0.136 ± 0.034, *p* = 0.008).

**Figure 2:**
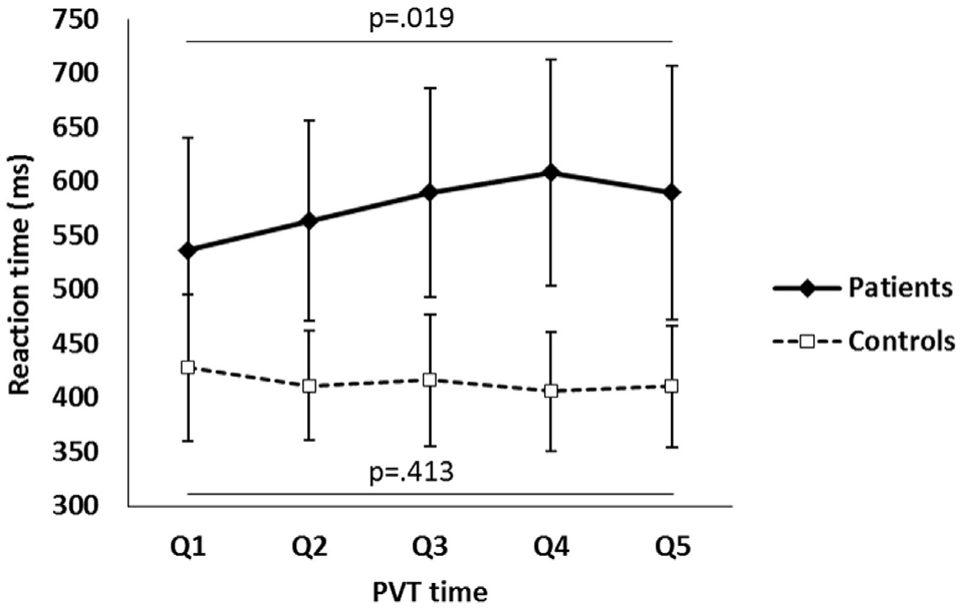
The mean reaction time (RT_mean_) in each quintile during the psychomotor vigilance task (PVT) performance for the mild traumatic brain injury patients and healthy control subjects. The error bars show the SD of RT (RT_std_) per quintile.

One-way ANOVA for repeated measures was performed to investigate if the performance time on test affected the changes in RT within subjects and between groups. Mauchly’s test indicated that the assumption of sphericity had been violated χ^2^ (9) = 42.125, *p* = 0.000, therefore degrees of freedom were corrected using Greenhouse-Geisser estimates of sphericity (ε = 0.41). The results showed that RT for patients and controls differed significantly (group effect) *F*(1.6, 29.63) as a function of time on task during PVT (*F* = 7.19, *p* = 0.005) (Figure 2). One-way ANOVA for repeated measures was also performed to investigate whether the performance time on task had a significant effect on the scatter in performance within subjects and between groups. Mauchly’s test indicated that the assumption of sphericity was not violated χ^2^ (9) = 13.174, *p* = 0.157. The results did not prove that the performance time on task had a significant effect on scattering in performance *F*(4, 72) = 1.6, *p* = 0.184.

The number of hours of sleep the night before the investigation was not associated to the RT_mean_ of PVT performance for both patients and controls. However, the hours of sleep was correlated with the RT_Std_ among the controls (*r*_con_ = −0.720, *p* = 0.029). No such correlation emerged for the patients (*r*_pat_ = −0.295, *p* = 0.408). There was no relation between self-rated fatigue (FSS) and sleep disturbances (PSQI) on the PVT for any of the groups.

### Correlation between Self-Rated Fatigue and Fatigability Measures

Table 1 shows correlation analysis results of fatigue and fatigability measures. Compared with RT_mean_, RT_Std_, and RT_RSD_ are significantly correlated (*p* ≤ 0.05) with self-rated fatigue in more number of quintiles and, therefore, are likely to be more sensitive measures of fatigability. For the controls, there is no single quintile where the self-rated VAS-f (pre-, post-PVT, and the percentage change) was significantly correlated with any of the fatigability measures, reflecting the fact there are no significant changes in fatigue and fatigability for the controls during PVT performance. Polling the data from both subject groups together improves the statistical power of the regression analysis, and there are more quintiles where the self-rated VAS-f are significantly correlated with fatigability measures (see Table 1, comparing the columns for “All” with the columns for mTBI). The correlations between VAS-f and fatigability measures are all positive indicating that higher fatigue is associated with longer RT and higher performance instability.

**Table 1:** Summary of regression analysis between self-rated fatigue and fatigability measures (RT_mean_, RT_Std_, and TR_RSD_) per quintile and total during the whole PVT.

		RT_mean_						RT_Std_						RT_RSD_					
Fatigue	Quintile	mTBI		Controls		All		mTBI		Controls		All		mTBI		Controls		All	
		*r*	*p*	*r*	*p*	*r*	*p*	*r*	*p*	*r*	*p*	*r*	*p*	*r*	*p*	*r*	*p*	*r*	*p*
Pre-PVT	Q1	0.48	0.17	0.13	0.72	0.44	0.05	0.53	0.12	0.13	0.72	0.45	0.05	0.57	0.08	0.09	0.80	0.35	0.13
	Q2	0.44	0.21	0.13	0.73	0.42	0.07	0.24	0.51	0.26	0.47	0.37	0.10	−0.1	0.78	0.2	0.59	0.25	0.29
	Q3	0.41	0.24	0.17	0.63	0.42	0.07	0.20	0.57	−0.08	0.83	0.25	0.30	−0.12	0.75	−0.10	0.79	−0.05	0.84
	Q4	0.45	0.20	−0.05	0.90	0.42	0.07	0.66	0.04	0.46	0.18	0.61	0.01	0.65	0.04	0.51	0.13	0.62	0.01
	Q5	0.44	0.21	0.27	0.46	0.43	0.06	0.33	0.35	0.54	0.11	0.46	0.04	0.18	0.63	0.57	0.09	0.47	0.04
	5Q	0.60	0.07	0.18	0.96	0.44	0.05	0.58	0.08	0.28	0.42	0.48	0.30	−0.62	0.05	0.04	0.90	0.05	0.83

Post-PVT	Q1	−0.23	0.52	0.33	0.35	0.42	0.07	−0.36	0.31	0.50	0.14	0.41	0.07	−0.29	0.41	0.39	0.27	0.34	0.14
	Q2	−0.24	0.51	0.19	0.61	0.38	0.10	−0.24	0.50	0.43	0.22	0.42	0.07	−0.19	0.60	0.38	0.27	0.44	0.05
	Q3	−0.22	0.55	0.15	0.68	0.40	0.08	−0.34	0.33	0.10	0.78	0.28	0.23	−0.28	0.43	0.09	0.82	0.07	0.76
	Q4	−0.20	0.59	0.26	0.47	0.44	0.05	−0.14	0.70	0.42	0.23	0.53	0.02	0.05	0.88	0.43	0.21	0.47	0.04
	Q5	−0.19	0.61	0.25	0.49	0.40	0.08	−0.22	0.55	0.48	0.16	0.45	0.05	−0.18	0.63	0.50	0.14	0.48	0.03
	5Q	−0.13	0.73	0.44	0.20	0.61	0.01	−0.20	0.58	0.53	0.12	0.61	0.04	−0.18	0.62	0.46	0.18	0.50	0.02

Normalized Fatigue change	Q1	−0.63	0.05	0.18	0.63	−0.21	0.38	−0.66	0.04	−0.02	0.97	−0.25	0.28	−0.59	0.07	−0.05	0.89	−0.20	0.39
	Q2	−0.59	0.07	0.15	0.68	−0.22	0.36	−0.38	0.28	−0.08	0.83	−0.19	0.42	0.02	0.95	−0.09	0.80	−0.10	0.68
	Q3	−0.54	0.11	0.04	0.91	−0.22	0.36	−0.33	0.35	−0.24	0.51	−0.24	0.32	0.11	0.75	−0.22	0.55	−0.16	0.51
	Q4	−0.56	0.09	0.33	0.35	−0.20	0.41	−0.76	0.01	−0.19	0.60	−0.33	0.16	−0.68	0.03	−0.25	0.50	−0.35	0.13
	Q5	−0.54	0.11	−0.02	0.96	−0.23	0.34	−0.54	0.11	−0.18	0.63	−0.26	0.28	−0.46	0.18	−0.19	0.59	−0.26	0.27
	5Q	−0.71	0.02	0.08	0.83	−0.15	0.52	−0.73	0.02	−0.18	0.96	−0.23	0.33	0.65	0.04	0.05	0.89	0.01	0.98

*Q1, Q2, … Q5 represent the results for the first, second, …fifth quintile, respectively. Here 5Q represents the results for the entire 20 min PVT performance period. mTBI, mild traumatic brain injury; PVT, psychomotor vigilance task*.

**Figure 3:**
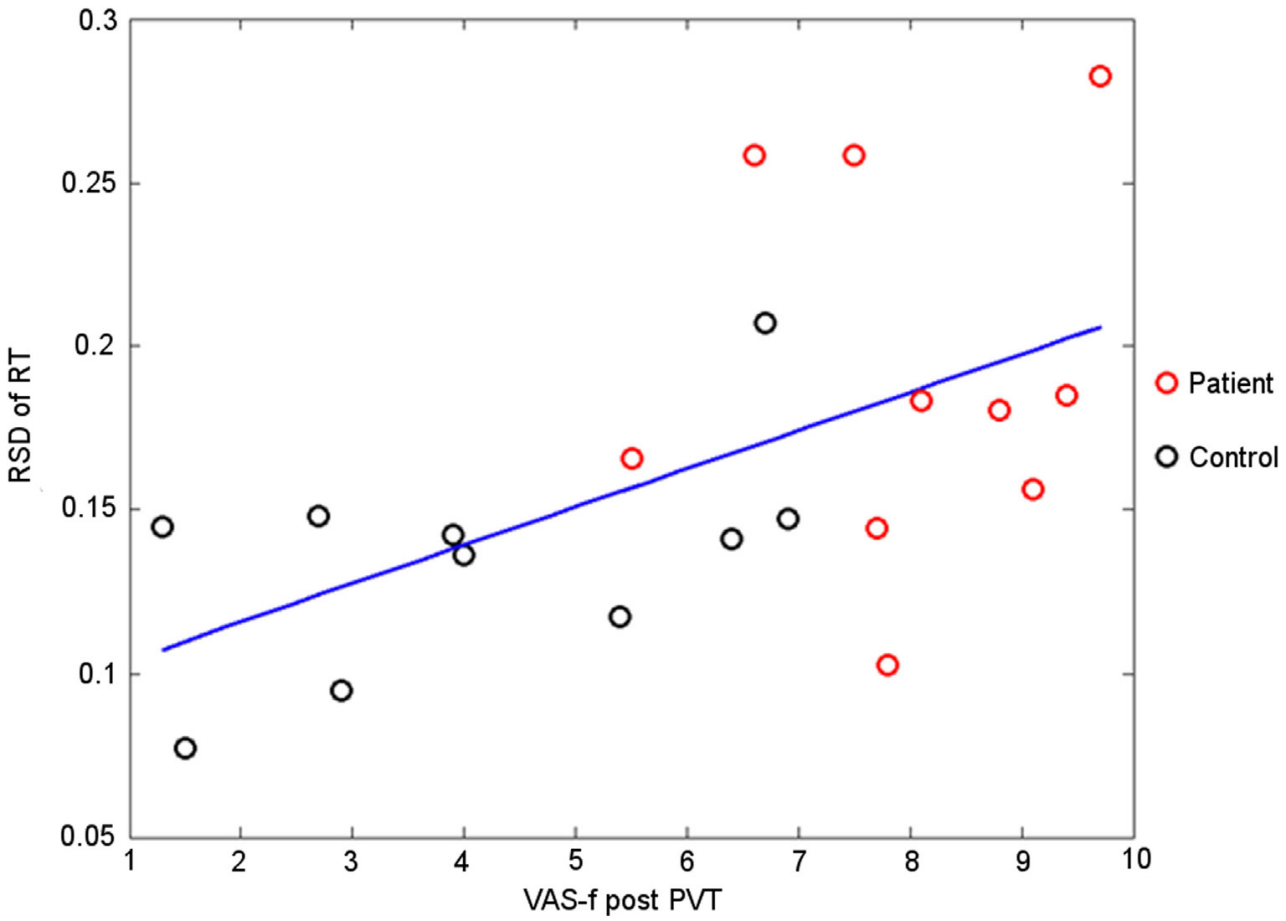
The RT_RSD_ in the last quintile of psychomotor vigilance task (PVT) performance vs. the self-rated fatigue immediate after the PVT performance. The line indicates the linear least square fitting to the measured data points (*r* = 0.48, *p* = 0.03).

As illustrated in Figure 3, the higher instability in cognitive performance was associated with higher fatigue complaint. The mTBI patients had not only higher self-rated fatigue than the healthy controls, but also higher fatigability as manifested by the RT_RSD_ (Figure 3).

Percentage changes from quintile 1 to quintile 5 in RT during PVT and percentage change on the VAS-f scale before and after MRI were also calculated. For the controls there was a significant correlation between difference in VAS-f rating pre- and post-PVT and the difference in mean performance during first and last quintile (fatigability) during the PVT (*r*_con_ = −0.830, *p* = 0.003), that is for the healthy controls the performance was in accordance with the evaluation of energy expenditure. This was not significant for the patients (*r*_pat_ = −0.455, *p* = 0.187).

### The Effects of PVT on Neural Activity

**Figure 4:**
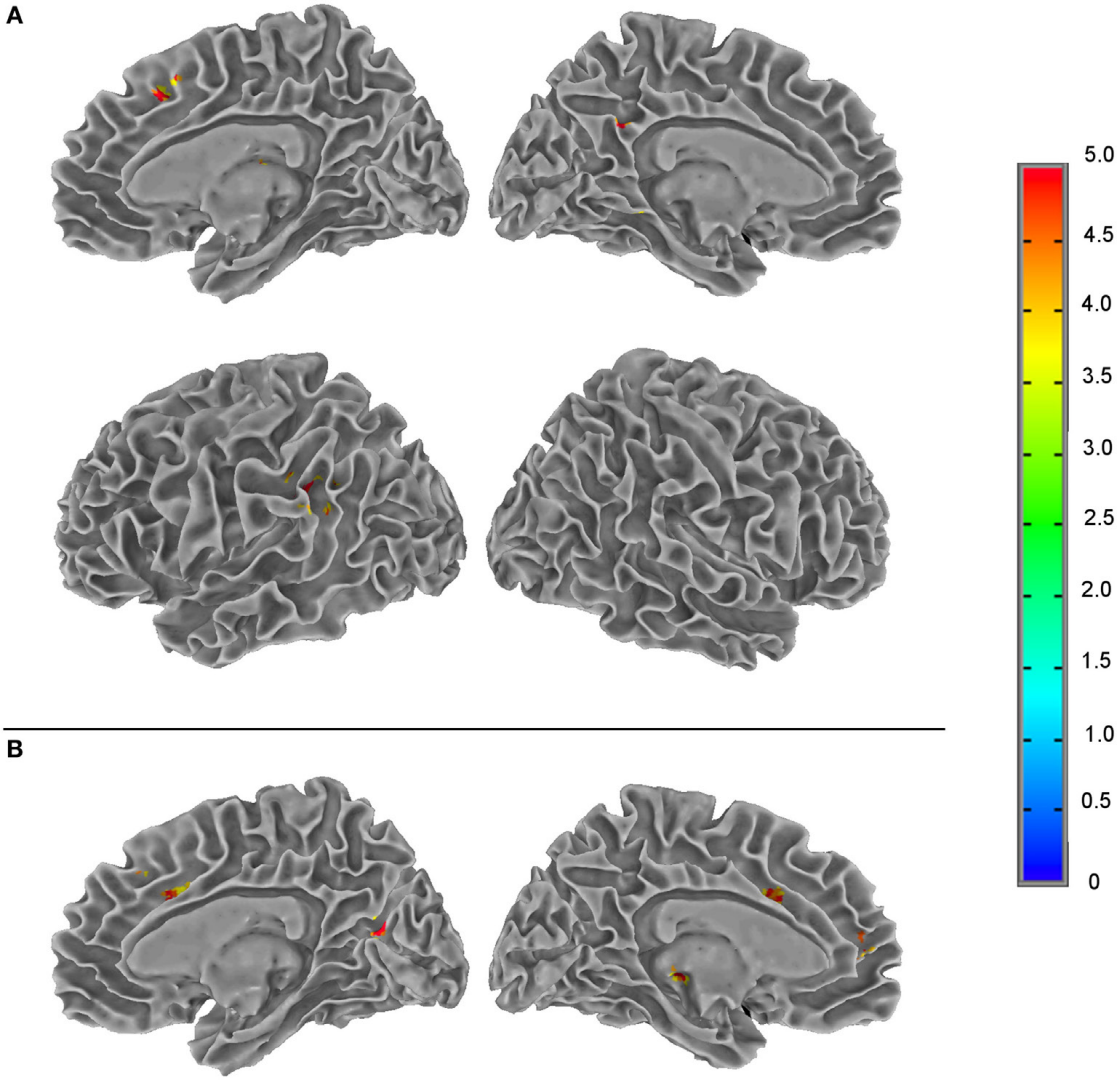
Summary of the *F*-score results from the three-way ANOVA modeling of the regional cerebral blood flow data acquired during a 20-min psychomotor vigilance task (PVT) performance to illustrate the brain regions of statistically significant differences (family-wise error rate, *p* ≤ 0.05) in neural activity associated with the two fixed factors (the PVT performance time and subject group) and their interaction. **(A)** The effect of PVT performance time; **(B)** the interaction effect between the PVT performance time and subject groups. The color bar indicates the *F*-score of the three-way ANOVA results.

The results from the three-way ANOVA modeling of the rCBF data acquired before and during PVT are summarized in Table 2 and Figure 4. There were significant rCBF changes (FWER, *p* < *0.05*) over the period of the PVT performance in left supramarginal gyrus, lentiform nucleus, precuneus, right thalamus and anterior cingulate cortex (ACC). For the individually normalized rCBF data over the entire PVT period, there was no significant group difference between mTBI patients and healthy controls when assessed with voxel-wise *p* < 0.01 in combination with a minimum cluster size ≥20. Significant interaction effects between the PVT performance time and subject groups (time × group) were detected in several brain regions including the right precuneus and insula, left thalamus and superior frontal gyri and left/right medial frontal gyri and ACC (see Table 2 and Figure 4).

**Table 2:** List of brain regions with statistically significant regional cerebral blood flow changes [family-wise error rate (FWER), *p* < 0.05] during sustained psychomotor vigilance task performance as identified by voxel-wise three-way ANOVA (retested measurements, model type = 5) by modeling time of performance and subject group as fixed factors and individual subject as the random factor.

Statistical contrast	ROI	Volume (voxel)	Location label	*x*, *y*, *z* (mm)	Brodmann area
Time	1	114	L-supramarginal gyrus	50, 46, 32	Left 40 (within 4 mm)
	2	49	L-lentiform nucleus	22, −10, 14	–
	3	23	L-precuneus	4, 50, 32	Left 31
	4	22	R-thalamus	−6, 18, 10	–
	5	22	R-anterior cingulate gyrus	−6, −24, 42	Right 32

**Group**	**–**				

Interaction (time × group)	1	54	R-precuneus	−8, 66, 16	Right 31 (within 2 mm)
	2	45	L-thalamus	10, 26, 0	–
	3	35	L-superior frontal gyrus	16, −32, 42	Left 8 (within 3 mm)
	4	30	L-medial frontal gyrus	6, −50, 8	Left 10
	5	26	R-anterior cingulate gyrus	−14, −24, 30	Right 32 (within 2 mm)
	6	26	R-medial frontal gyrus	−6, −32, 36	Right 9
	7	24	L-anterior cingulate gyrus	10, −14, 34	Right 24 and 32 (within 1 mm)
	8	24	R-insula	−44, 12, 16	Right 13 (within 1 mm)

*Here x, y, and z represent the peak locations of the detected clusters in the standard MNI coordinates. The assignments of Brodmann areas for the peak locations were also specified. FWER was assessed with a minimum cluster size of 20 contiguous voxels and a voxel-wise p < 0.01*.

With voxel-wise regression analysis of rCBF data with RT data, we detected significant positive correlation for the mTBI patients between RT_mean_ and rCBF during quintile 5 of the PVT performance in the right middle frontal gyrus (*r*_pat_ = 0.851, *p* = 0.001). We could also see significant correlation for the patients between RT_std_ and rCBF during quintile 2 in left middle frontal gyrus (*r*_pat_ = 0.882, *p* = 0.000).

**Figure 5:**
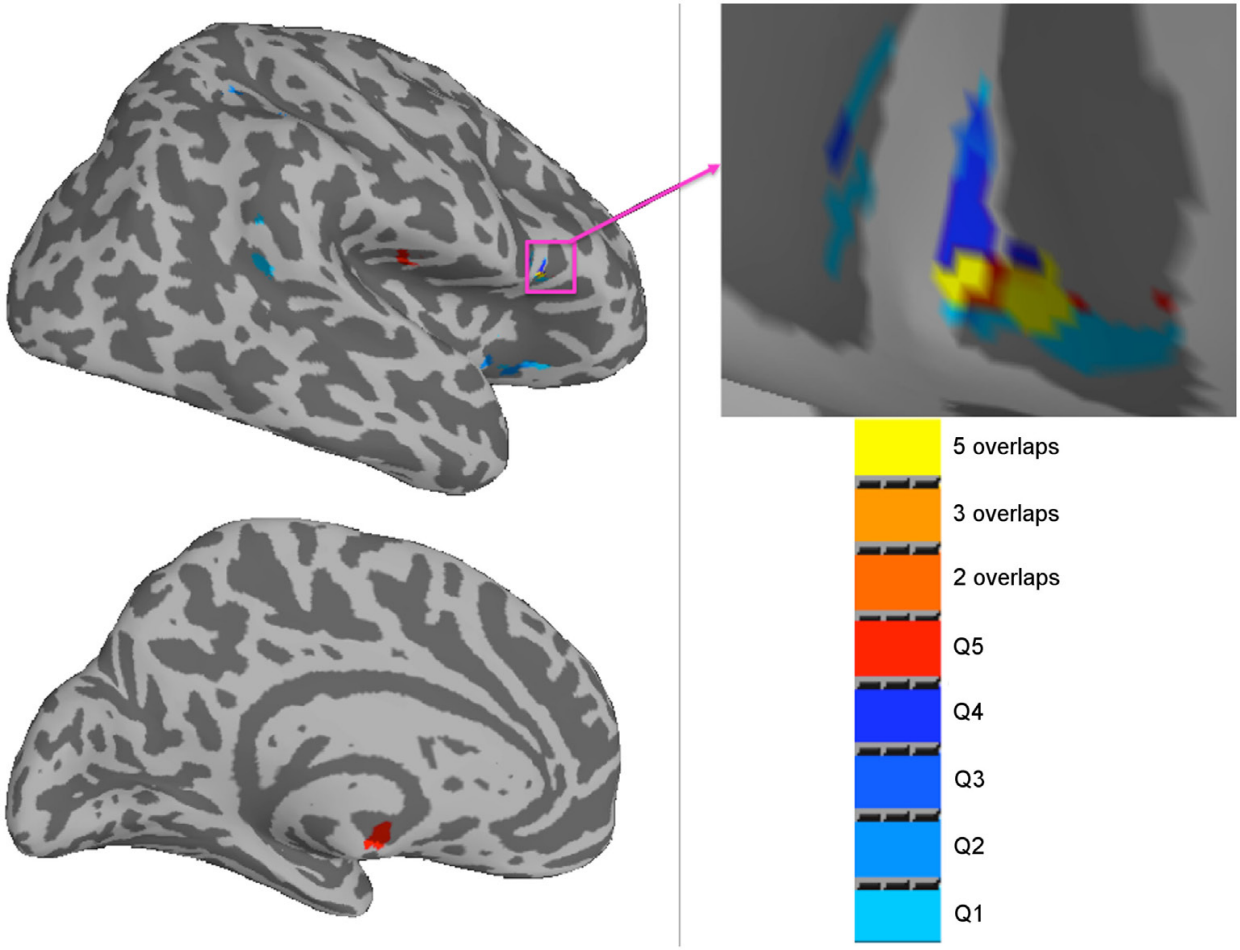
Summary of brain regions with a significant correlation (family-wise error rate, *p* < 0.05) between regional cerebral blood flow and the Visual Analog Scale of Fatigue ratings after MRI for mild traumatic brain injury patients (*n* = 10) in each quintile of the psychomotor vigilance task performance. The color bar indicates the results from the different quintiles and their overlaps.

Voxel-wise regression analyses were performed between rCBF during PVT and the VAS-f ratings. Significant correlations were found between rCBF and the VAS-f ratings after MRI in several brain regions for the patients (Table 3), but no significant correlations emerged for the controls. The brain regions reported in Table 3 are visualized in Figure 5. No significant correlations were found between VAS-f change or VAS-f before PVT.

**Table 3:** List of brain regions with statistically significant correlation [family-wise error rate (FWER), *p* < 0.05] between the normalized regional cerebral blood flow and Visual Analog Scale of Fatigue ratings after MRI for the mild traumatic brain injury patients during each quintile of the psychomotor vigilance task performance.

Time	Location label (Brodmann)	*x*, *y*, *z*	Volume (voxel)	*p*-Value	*r*
Q1	R-anterior cingulate (32)	−18, −40, 2	49	0.000	−0.906
	R-inferior frontal gyrus (47)	−32, −20, −16	31	0.001	−0.853
	R-lentiform nucleus	−12, 6, −4	25	0.001	−0.864
	R-precentral gyrus (44)	−54, −10, 6	24	0.000	−0.894

Q2	R-precentral gyrus	−52, −12, 10	29	0.000	−0.898
	R-lentiform nucleus/R-putamen	−24, −2, 10	29	0.000	−0.920
	L-inferior parietal lobule	34, 46, 46	23	0.000	−0.905
	R-superior temporal gyrus	−52, 46, 16	20	0.000	−0.896

Q3	L-medial frontal gyrus	16, −56, 16	55	0.001	−0.868
	R-inferior frontal gyrus	−28, −26, −18	28	0.000	−0.873
	R-inferior frontal gyrus (47)	−56, −12, 4	25	0.000	−0.906
	R-lentiform nucleus/R-putamen	−30, 4, 6	21	0.001	−0.846
	R-inferior parietal lobule (40)	−38, 48, 54	20	0.000	−0.888

Q4	R-superior temporal gyrus	−56, −12, 4	29	0.000	−0.900
	L-superior frontal gyrus	18, −60, 16	28	0.000	−0.920

Q5	L-anterior cingulate (32)	16, −44, 4	66	0.000	0.940
	L-insula	26, 20, 22	33	0.000	−0.901
	L-thalamus	0, 14, −10	31	0.000	−0.909
	R-caudate	−18, −2, 24	30	0.000	−0.879
	R-precentral gyrus	−48, 14, 14	24	0.000	−0.905
	L-caudate	14, −22, 14	22	0.000	−0.892
	L-medial frontal gyrus	6, −52, −12	20	0.000	0.892
	R-precentral gyrus	−50, −10, 6	20	0.000	−0.880

*Here x, y, and z represent the peak locations of the detected clusters in the standard MNI coordinates. FWER was assessed with a minimum cluster size of 20 contiguous voxels and a voxel-wise p < 0.01. The assignments of Brodmann areas for the peak locations were also specified*.

Voxel-wise regression analyses were also performed between the rCBF and the corresponding RT_RSD_ during each quintile of the PVT performance. The regression analyses revealed significant correlations in left lingual gyrus (Q1, Brodmann 18), right middle temporal gyrus (Q2, Brodmann 21), right inferior frontal gyrus (Q4, Q5, Brodmann 47/45), left superior frontal gyrus (Q4, Q3, Brodmann 22), right superior frontal gyrus (Q5, Brodmann 22), and left middle and superior temporal gyrus (Q5).

The additional voxel-wise *t*-tests did not reveal any significant group differences in the rCBF data. The average whole-brain rCBF (mean ± SD/ml/100 g/min) for the mTBI patient group at rest and during PVT were 65.6 ± 8.5 and 68.3 ± 7.1, respectively. For the control group, the corresponding rCBF values were 63.4 ± 11.8 (at rest) and 65.4 ± 10.3 (during the PVT). There was no significant difference in whole-brain rCBF between the mTBI patient and healthy control groups at rest or during PVT performance. There was no significant correlation between the whole-brain rCBF vs. RT, RT_Std_, or RT_RSD_.

## Discussion

In our study, we aimed at investigating the relationship between the dynamic changes of neural activity and cognitive fatigability during a 20 min PVT and the self-rated fatigue score before and after the PVT. The significant interaction effect (time × group) detected by ANOVA modeling of rCBF data measured during PVT indicate that mTBI patients suffering from chronic fatigue processed the task differently compared to healthy controls. During the PVT the mTBI patients had an altered rCBF in several regions including left thalamus and superior frontal gyri, right precuneus and insula, and left/right medial frontal gyri and ACC, compared to the healthy controls. These regions have been associated with fatigue in previous studies (19, 28–31).

A neuroanatomical model of fatigue has gradually begun to emerge as imaging research has developed new methods. Chaudhuri and Behan were early to realize the importance of the non-motor functions of basal ganglia for explaining mental fatigue (32), but structures in the basal ganglia and thalamus were not sufficient to explain the model. Later a network of brain regions including also the middle frontal gyrus, ACC, orbitofrontal cortex, insular cortex, amygdala, superior parietal cortex, and nucleus accumbens have been associated with fatigue (19, 28–31). In lesion studies by Picton and coworkers a network involving the superior medial region of the frontal lobe and ACC has been linked to energizing in attention demanding task (33, 34). Disturbances in these areas might result in slow performance time in attention tasks that require the ability to sustain preparation and readiness to respond for procedures that are not highly overlearned (33). In the present study, the frontal regions with altered rCBF during sustained PVT coincide with areas of the brain which were suggested to be related to energizing, i.e., the process of initiating and maintaining a response (35). We observed also enhanced neural activity in the right middle frontal gyrus that was strongly related to slow performance at the end of the PVT among the patients, but not the controls.

In this study, we also found that self-rated fatigue was related to functional activity in some other structures in the brain. We found no significant correlation between RT_mean_ and rCBF in the ACC, but we did find strong associations between self-rated fatigue post-PVT and rCBF in the ACC. Among the patients, high post-PVT fatigue was correlated with initial (first quintile) low activity in the right ACC and at the end of the task (in the fifth quintile), high activity in the left ACC and the medial frontal gyrus, indicating reduced performance monitoring and monitoring functions of ACC as a result of impaired cognitive control (36). This shows an interesting process as performance on PVT and self-rated fatigue were positively correlated. However, it should be noted that even high performers among the patients scored in the lower range when compared with controls. The inability to predict a magnitude of energy demands for the required performance has been proposed as central in mental fatigue (31, 37). It appears that the mTBI patients with chronic fatigue have impaired ability to evaluate or adapt to the energy demands of the sustained PVT performance and can lead easily to high self-rated fatigue. This is also correlated to the alterations of rCBF in ACC and medial frontal gyrus during PVT performance. The mTBI patients lacked energizing capacity as manifested by increased fatigability during time-on-task. Also, they might have a decreased ability to evaluate their mental effort, i.e., gauging the relationship between performance over time and fatigue.

Our study strengthens earlier proposals (11, 38) that we need to distinguish between fatigue and fatigability. Cognitive fatigability seems to be more related to the energization systems in the brain that coincide with areas referred as superior medial circuits (39) while self-rated fatigue is more related to an inability to adapt to the lack of internal energy. The middle frontal gyrus was related to slow reaction time at the end of the PVT, indicating an influence on the energization system, while the medial frontal gyrus reemerged in time and group effects of cerebral blood flow, and was also related to self-rated fatigue after the PVT. The medial frontal gyrus seems, therefore, to be a core structure for a combination of both performance-related fatigability and self-rated fatigue. However, self-reported fatigue after the PVT was also consistently related to low activity in the right inferior frontal gyrus among the patients. Whether the networks for fatigability and self-rated fatigue are different, needs, hence to be investigated in future larger studies.

The performance of the patients was more scattered compared to the healthy controls, indicating their problems with monitoring a stable performance level. However, the degree of scattering in performance did not increase over time. For the healthy controls scattered performance level was related to fewer hours of sleep the night before, in line with earlier studies on the effect of reduced sleep on vigilance performance (40) and brain connectivity (41). This relation was not found among the patients. Individual performance variability and performance speed have been associated to the right inferior frontal gyrus in previous studies (42)—a region that also has been associated with frontal control when performing go/no-go tasks (43) and alterations when performing fatigue-inducing mental tasks (44). In the present study, at the *p* = 0.05 level, we found group differences in CBF in the right inferior frontal gyrus, suggesting that the scattered performance among patients probably were more related to altered CBF in inferior frontal gyrus rather than sleep disturbances.

We also found a correlation between scattered PVT performance (RT_RSD_) and functional activity in the visual and temporal regions. One possible explanation is that the patients’ discrete visual disturbances (45) can lead to increased performance strain and instability. The relationship between visual impairment and fatigue in mTBI needs to be further studied.

Mental fatigue is a broad concept where cognitive fatigability, i.e., performance decrease over time, may be more related to the brain’s energizing system while scattered performance rather seems to be related to cognitive control and a subjective feeling of fatigue. In clinical settings, this means that patients suffering from mental fatigue could be expected to perform differently on neuropsychological tasks depending on the localization of injury—still tests including a higher level of mental control in combination with performance speed are expected to be influenced (5, 16).

To minimize cortical responses related to the handedness of the study participants, we instructed all participants to use their dominant hand for the PVT response. Increased activation has previously been shown in sensorimotor areas following the use of non-dominant hand performing motor tasks during fMRI acquisition (46). Learning effects have been demonstrated to decrease rCBF changes while performing a complicated motor task using positron emission tomography (47). The PVT presented in our study comprised a relatively simple motor task since only one button response was used. The 2-min training session performed by all participants before MRI/PVT is very likely to contribute to some learning effect for such a simple task. Therefore, we expect minimal contribution from the motor part of the PVT to the functional results we report.

A limitation of the present study is the low number of participants, but the results are consistent with findings from the earlier studies on fatigue (19, 28, 29, 48). The subjects in each group were rather homogeneous without confounders, such as the presence of psychiatric conditions, history of earlier brain pathology, and substance abuse. However, a larger sample size would be warranted for more robust statistics. Another limitation is that the PVT quintiles were divided according to the number of responses during the task, while the rCBF quintiles were divided into strict 4 min intervals. This means that the temporal convergence was not perfect, which may have affected the correlation between rCBF and PVT. However, both PVT and rCBF measurement lasted exactly 20 min, the temporal difference was estimated to be relatively small and it was not systematic. However, future studies should measure time intervals on the PVT test. We used a study design to minimize inter-subject variations in global rCBF contributing to noise in group comparisons, associated with non-neural factors such as breathing pattern, physiologic state, and caffeine consumption (49). We normalized the rCBF data during the PVT to the resting-state rCBF before the onset of the task to minimize inter-subject differences in the baseline rCBF. By performing normalization, the statistical power can thus be improved. Characteristics of the PVT might have also influenced the results [Drummond et al. (50)]. In most PVT tasks, stimuli are presented at random intervals of 2–10 s (19, 50). Inter-stimulus intervals of 2–10 s are adequate when designing a vigilance task by “keeping subjects on their toes,” but they are insufficient to reflect the actual path of the hemodynamic processes (50). The visual feedback displayed after each response might have also contributed to arousal maintenance. The influence of these parameters needs to be investigated in further studies.

## Conclusion

The significant findings from this study are: (1) compared to healthy controls, patients suffering from chronic fatigue following mTBI show not only higher self-rated fatigue but also cognitive fatigability manifested as progressively slower, unstable, and scattered performance during a 20 min PVT; (2) RT_Std_ and RT_RSD_ are more sensitive measures of cognitive fatigability than RT_mean_ and are more closely correlated with self-rated fatigue; (3) fatigability among the patients was associated with different activity changes during performance compared to what was seen in healthy controls. (4) Self-rated fatigue and cognitive fatigability are associated with somewhat different functional networks, but this needs to be further confirmed in future studies with larger sample sizes. (5) The results of this study demonstrate that PCASL is a useful technique to investigate neural correlates of fatigability during a sustained PVT. The findings from the present study confirm further the results from previous ASL study of healthy volunteers (19) and resting-state fMRI study of mTBI patients (17). Measurement of rCBF and quantitative mapping of functional connectivity can provide an objective assessment of fatigability during a sustained PVT. PCASL measurement and resting-state BOLD fMRI hold promise to provide potential biomarkers for evaluating chronic fatigue in mTBI patients.

## Ethics Statement

This study was carried out in accordance with the recommendations of the regional ethics committee in Stockholm. All subjects gave written informed consent in accordance with the Declaration of Helsinki.

## Author Contributions

MM: input to the idea and design of the study, recruitment of patients, assisting the fMRI investigation, data processing and statistical evaluation and interpretation of the PVT and demographic variables, and manuscript conceptualization and writing; LN: programming and major data processing of the PVT, performing the fMRI investigations, statistical evaluation and interpretation of the fMRI and PVT data, manuscript conceptualization and writing; AB: idea and design of the study, contributed to interpretation of the findings and manuscript writing, PJ: idea and design of the study, contributed to interpretation of the findings and the manuscipt; T-QL: design of the study and the fMRI investigations, major statistical evaluation and interpretation of the fMRI and PVT data, and major contribution to the manuscript. All authors have approved the final version of the paper and agree to be accountable for all aspects of the work in ensuring that questions related to the accuracy or integrity of any part of the work are appropriately investigated and resolved.

## Conflict of Interest Statement

The authors declare that the research was conducted in the absence of any commercial or financial relationships that could be construed as a potential conflict of interest.
